# Genetic diagnosis of Jordanian patients with glycogen storage diseases

**DOI:** 10.1186/s13023-025-03982-z

**Published:** 2025-10-03

**Authors:** Mohammad Shboul, Mohammed El-Khateeb, Rajaa Fathallah

**Affiliations:** 1 Department of Medical Laboratory Sciences, Faculty of Applied Medical Sciences, Jordan University of Science and Technology, P.O. Box 3030, Irbid, 22110 Jordan; 2 National Center for Diabetes, Endocrinology and Genetics, Amman, Jordan

**Keywords:** Metabolic, Variants, Whole exome sequencing, Gene, Glycogen storage diseases

## Abstract

**Background:**

Glycogen storage diseases (GSDs) are a group of hereditary metabolic disorders caused by defects in biosynthesis, and storage of glycogen that affect various organs, such as liver, muscles, and heart. Approximately 29 genes are implicated in GSDs. This study aimed to identify the genetic variants causing GSDs in Jordanian patients.

**Methods:**

Twenty patients with clinically suspected GSD were studied. Clinical data were reviewed and whole exome sequencing (WES) was conducted. Variants were validated, and the carrier status of the parents was confirmed by Sanger sequencing. Following genetic counseling, seven families opted for prenatal diagnosis (PND), and one of them proceeded with preimplantation genetic diagnosis (PGD) after the previous unsuccessful PND.

**Results:**

Thirteen putative disease-causing variants in nine genes (*AGL*, *GAA*, *GBE1*, *G6PC*, *PHKA2*, *PHKB*, *PHKG2*, *SLC2A2*, and *SLC37A4*) were identified. Three of these variants have never been published. GSDII was the most common type (25%), followed by GSDIa (20%), GSDIb (15%), GSDIII and GSDIXC (10% each), and GSDIV, GSDIXa, GSDIXb, and GSDXI (5% each). Out of eight PND cases, two fetuses were affected, whereas the rest were unaffected. PGD identified two normal embryos out of the 13 tested, resulting in the successful birth of a healthy son.

**Conclusions:**

This study expands the genetic spectrum of GSD-associated genes and highlights the role of consanguinity in disease prevalence. WES has proven effective for the diagnosis of GSDs, facilitating accurate disease identification, early treatment, and management. These findings are valuable for genetic counseling, PND, and PGD as effective tools for disease prevention and family planning.

**Supplementary Information:**

The online version contains supplementary material available at 10.1186/s13023-025-03982-z.

## Background

Glycogen storage diseases (GSDs) are heterogeneous metabolic disorders with overlapping phenotypes that affect the biosynthesis and storage of glycogen resulting in the accumulation of glycogen in several tissues, such as the muscles, liver, and heart, in addition to the kidneys, intestine, and central nervous system [1, 2]. The metabolism of glycogen is regulated by a group of enzymes and defects in any of them cause several GSD types. To date, over 20 distinctGSD types including subtypes have been classified according to the disrupted genes and encoded enzymes as well as affected tissues and clinical features [3–5]. These disorders can be categorized as liver-predominant (GSD types 0, I, III, IV, VI, IX, XI), muscle-predominant, or both [4, 6, 7]. The muscle GSDs can be further subdivided into those presenting with exercise intolerance but no cardiac defects (GSD V, VII, X, XI, XII, XIII, XIV and phosphoglycerate kinase deficiency) and those associated with cardiac manifestations (GSD II, IIb, III).

However, most GSDs result from defective cytoplasmic enzymes, Pompe disease is caused by a deficiency of a lysosomal enzyme (acid α-glucosidase) that leads to abnormal accumulation of glycogen in cardiac and skeletal muscles, therefore it is also classified under lysosomal storage diseases [4]. In some types of GSDs, other organs including kidney and brain can also be affected, which further complicating classification and management. Neonatal- and infantile-onset GSDs tend to have more severe clinical presentations, whereas milder types may remain asymptomatic or present later in life [2].

Among the known GSDs, GSD type IV (Andersen disease), is a clinically heterogenous disorder caused by deficiency of glycogen branching enzyme 1 (GBE1). This enzyme is essential for the formation of normal branched structure of normal glycogen. The deficiency of GBE1 leads to the accumulation of polyglucosan bodies, abnormal glycogen polymers with reduced branching structure that are insoluble and resistant to normal degradation unlike the normal glycogen [9]. These abnormal structures are cytotoxic and interfere with normal cellular function, contributing to progressive tissue dysfunction.

GSD IV is classified into six clinical subtypes, based on clinical manifestations, severity and age of onset. These include two hepatic forms: the severe “classic-progressive” and the milder “non-progressive” as well as four neuromuscular forms including in-utero onset neuromuscular, congenital neuromuscular, childhood neuromuscular, and adult polyglucosan body disease [8, 9]. The broad phenotypic spectrum of the disease reflects the diverse roles of GBE1 in different tissues and underscores the complexity of diagnoses and management GSD IV.

The early diagnosis of GSDs is critical for enhancing the quality of life of afflicted patients and allowing for suitable therapeutic approaches [5]. Furthermore, understanding the genetic foundations of GSD patients can aid in their families’ counseling. However, the clinical diagnosis of a specific GSD type can be difficult, genetic approaches provide more efficient diagnostic tools, and there is still potential for improvement in terms of speed and cost-effectiveness.

Advances in next-generation sequencing (NGS) during the last decade have made whole exome sequencing (WES) feasible for the diagnosis of genetic diseases including GSDs [5, 10, 11].

In Jordan, GSDs are rare and have only been reported in the literature as case studies [12, 13]. In this study, we performed WES to identify the genetic basis of 20 Jordanian patients with suspected GSD. We successfully identified the causative variants in all patients. In total, 13 variants were identified in GSD-associated genes.

## Methods

### Subjects and ethics statement

Twenty patients who were suspected of having GSD were enrolled in this study. Patients were recruited from the National Center for Diabetes, Endocrinology and Genetics, and private clinics. The study was approved by the Ethical Committee of Jordan University of Science and Technology. Written informed consent was obtained from all the families.

### Samples and genomic DNA extraction

Peripheral blood samples were also collected from all patients and available family members. The genomic DNA was extracted using Bio Robot EZ1 following the manufacturer’s instructions (Qiagen, Solna, Sweden). The quality and concentration of the DNA were assessed via a Nanodrop 2000 C spectrophotometer (Thermo Fisher Scientific, Waltham, MA, USA) and 1% agarose gel electrophoresis.

### Whole exome sequencing (WES) and variant analysis

An Agilent’s SureSelect Human All Exon V6 Kit was used following the manufacturer’s instructions. The final indexed libraries were sequenced to 100X coverage with 2 × 150 chemistry on the Illumina NovaSeq5000 platform (Illumina, United States). Approximately 24,000 genes were sequenced, with exome coverage (> 10X) > 98%. Sequence reads were aligned to the human reference genome (GRCh37/hg19). Clinically relevant variants were annotated on the basis of available information from the literature and a set of databases, such as the HGMD (http://www.hgmd.cf.ac.uk), 1000 Genomes (http://www.1000genomes.org), ClinVar (http://www.ncbi.nlm.nih.gov/clinvar), and gnomAD v4.0.0 (https://gnomad.broadinstitute.org/), in addition to an in-house database. Variants with a minor allele frequency (MAF) of more than 1% in the gnomAD database were filtered out. Missense, frameshift, nonsense, and typical splice site variants (+/-20 intronic bases) were prioritized. Several in-silico tools have been used to evaluate the effects of identified variants on protein function [14]. Splice site variants were analyzed by splice AI (https://spliceailookup.broadinstitute.org/). All variants were classified according to the guidelines of the American College of Medical Genetics and Genomics (ACMG) [15].

### Validation of the identified variants

Sanger sequencing was used to validate the identified variants in families using the BigDyeTM Terminator v3.1 Cycle Sequencing Kit on the ABI Prism 550 automated sequencer (Applied Biosystems, Foster City, CA).

## Results

### Clinical description of patients

Twenty patients (9 males and 11 females) from 20 unrelated families were recruited for this study. The age, sex, age of onset and clinical manifestations at the time of diagnosis are summarized in Table 1. The age of diagnosis ranged from 1 month to 13 years. All affected children were alive at the time of diagnosis. Consanguinity was observed in 19 out of 20 families (95%). A metabolic screen was not performed for these patients because of financial constraints. Therefore, the definitive clinical diagnosis is unknown.

**Table 1:** Clinical features of patients with glycogen storage diseases

Family	Patient	Sex	Age of diagnosis	Liver	Muscle	Additional finding	Degree of relationship^a^
F1	P1	F	1 year	+	−	Hypotonia, hypoglycemia	First cousins
F2	P2	M	7 months	+	+	lactic acidosis, elevated liver enzymes, hypokalemia, hypercalcemia, hypoglycemia	Not relatives
F3	P3	F	7 years	+	−	Hypoglycemia, neuropathy, raised uric acid and lactic acidosis	First cousins
F4	P4	F	< 1 month	+	−	Seizures, hypoglycemia. A history of one affected relative	Second cousins
F5	P5	F	< 1 month	+	−	Hypoglycemia, short stature. A history of two affected brother and sister who died at the age of 5 year	First cousins
F6	P6	M	10 months	+	−	Hypoglycemia, oral ulcer, distended abdomen and seizure, died at the age of 1 year due to lung and throat inflammation and mouth infection	First cousins
F7	P7	M	18 months	+	−	Hypoglycemia, distended abdomen, oral ulcers, chronic diarrhea, seizures and elevated immunoreactive trypsinogen	First cousins
F8	P8	M	5 months	−	−	Hypertrophic cardiomyopathy, hypotonia, died at age of 7 months	First cousins
F9	P9	M	5 months	NA	NA	NA	First cousins
F10	P10	F	6 months	+	+	Muscle weakness, hypotonia, hypertrophic cardiomyopathy, died at age of 8 months	Second cousins
F11	P11	F	5 months	+	+	Muscle weakness, hypotonia, hypertrophic cardiomyopathy, died at age of 6 months	First cousins
F12	P12	F	5 months	−	+	Hypertrophic cardiomyopathy, muscle weakness for one girl. A history of affected sister	First cousins
F13	P13	M	2 years	+	−	A history of affected cousins	First cousins
F14	P14	F	2 years	+	−	A history of affected sister	First cousins
F15	P15	F	16 months	+	−	Hepatosplenomegaly, died at age of 2 years	First cousins
F16	P16	M	6 months	−	−	Diarrhea, fever, distended abdomen, neutropenia, thrombocytopenia, bone marrow aspirate showed severe dysplastic	First cousins
F17	P17	M	13 years	+	−	Elevated liver enzymes	First cousins
F18	P18	F	9 years	+	−	Elevated liver enzymes, metabolic acidosis. A history of affected brother and cousins	First cousins
F19	P19	M	1 year	+	−	Elevated liver enzymes, metabolic acidosis	First cousins
F20	P20	F	2 years	+	−	Fanconi syndrome, hypoglycemia, immunodeficiency, distended abdomen, bronchitis, gastroenteritis, stomatitis, recurrent infections, elevated liver enzymes, triglyceride, cholesterol, potassium, lactic acid, ammonia, parathyroid hormone, positive urine ketones, increased prothrombin time, elevated microalbuminuria.	First cousins

^a^Parents relationship.

Abbreviations: GSD, glycogen storage disease; F, female; M, male; NA, not available

The most common chief complaints were hepatomegaly or abdominal distension (16 patients), hypoglycemia (8 patients), elevated liver enzymes (5 cases), hypertrophic cardiomyopathy (3 patients), and short stature or hepatosplenomegaly (1 patient for each) (Table 1).

The clinical descriptions are summarized as follows: Patient (P1) presented with hypoglycemia, hepatomegaly, and hypotonia. Patient (P2) presented with lactic acidosis, hepatomegaly (without splenomegaly), hypoglycemia, elevated liver enzymes, hypokalemia, hypercalcemia, and muscle weakness. Patient (P3) exhibited hepatomegaly, hypoglycemia, and lactic acidosis. Patient (P4) had hepatomegaly, hypoglycemia, and seizures. Patient (P5) had short stature, hepatomegaly, and hypoglycemia. Patient (P6) experienced recurrent hypoglycemia since birth, hepatomegaly and a distended abdomen. He died at the age of 1 year due to lung and throat inflammation and mouth infection. Patient (P7) presented with hepatomegaly, hypoglycemia, a distended abdomen, chronic diarrhea, and seizures. Patients (P8) had severe hypertrophic cardiomyopathy and hypotonia and died at the age of 5 months. No clinical descriptions were available for the patient (P9).

Patients (P10 and P11) are unrelated children, presented with muscle weakness, hypotonia, and hypertrophic cardiomyopathy. They died at the ages of 8 months and 6 months, respectively. Patient (P12) presented with muscle weakness and hypertrophic cardiomyopathy and had an affected sister suffering from hypertrophic cardiomyopathy. Both P12 and her sister are alive and are currently receiving enzyme replacement therapy (ERT). Patients (P13 and P14) are unrelated children who presented with hepatomegaly and had a family history of affected relatives.

Patient (P15) presented with hepatosplenomegaly and died at the age of 2 years. Patient (P16) presented with severe diarrhea, fever, and abdominal distention. A complete blood count showed neutropenia and thrombocytopenia, whereas bone marrow aspirate revealed severe dysplasia. This patient also had low serum folic acid levels. Hypoglycemia and hepatomegaly were not observed at the time of diagnosis. Patient (P17) presented with hepatomegaly and elevated liver enzymes. Patients (P18 and P19) are unrelated children with elevated liver enzymes, and metabolic acidosis. In the last patient (P20), Fanconi-Bickel syndrome (GSDXI) was suspected. The patient presented with hepatomegaly, hypoglycemia, immunodeficiency, abdominal distension, bronchitis, gastroenteritis, stomatitis, and recurrent infections, as well as elevated liver enzymes, triglycerides, cholesterol, potassium, lactic acid, and ammonia.

### Genetic analysis results

By whole exome sequencing (WES), we identified 13 variants in different GSD-associated genes (Table 2). Nine types of GSDs were identified in this study. Among these, GSDII was the most common type (5/20, 25%) of GSD followed by GSDIa (4/20, 20%), GSDIb (3/20, 15%), GSDIII and GSDIXC (2/20, 10% each), and GSDIV, GSDIXa, GSDIXb, and GSDXI (1/20, 5% each).

**Table 2:** Variants identified by whole exome sequencing in nine different genes associated with glycogen storage diseases

#	Gene RefSeq	Variant						GSD type	Inheritance	Cases
		Chromosome^a^ Coding Protein	dbSNP ID	Allele frequency (gnomAD)	Type	ACMG	Zygosity			
1	*G6PC* NM_000151.4	Chr17-41055964C>T c.247C > T p.(Arg83Cys)	rs1801175	0.006557	Missense	P	Hom	GSDIa	AR	P1-P4
2	*SLC37A4* *NM_001164277.2*	Chr11-118895980CAG >C c.1042_1043del p.(Leu348Valfs*53)	rs80356491	0.0003431	Frameshift	P	Hom	GSDIb	AR	P5-P7
3	*GAA* *NM_000152.5*	Chr17-78081378CT>C c.716del p.(Leu239Argfs*29)	rs1555599594	NA	Frameshift	P	Hom	GSDII	AR	P8
4	*GAA* *NM_000152.5*	Chr17-78086427C>T c.1805C > T p.(Thr602Ile)	NA	NA	Missense	LP	Hom	GSDII	AR	P9
5	*GAA* *NM_000152.5*	Chr17-78086796CAT>C c.2011_2012del p.(Met671Alafs*65)	NA	NA	Frameshift	LP	Hom	GSDII	AR	P10
6	*GAA* *NM_000152.5*	Chr17-78086801G>A c.2015G>A p.(Arg672Gln)	rs778418246	0.0001688	Missense	P	Hom	GSDII	AR	P11, P12
7	*AGL* *NM_000642.3*	Chr1-100340911C>G c.1186-3C>G	NA	NA	Splice site	VUS	Hom	GSDIII	AR	P13
8	*AGL* *NM_000642.3*	Chr1-100356892C>T c.2929 C>T p.(Arg977*)	rs531425980	0.000007761	Nonsense	P	Hom	GSDIII	AR	P14
9	*GBE1* *NM_000158.4*	Chr3-81691938T>C c.986 A>G p.(Tyr329Cys)	rs80338671	0.0008828	Missense	LP	Hom	GSDIV	AR	P15
10	*PHKA2* *NM_000292.3*	ChrX-18,942,552C>T c.1661G>A p.(Trp554*)	NA	NA	Nonsense	P	Hemi	GSDIXa	XLR	P16
11	*PHKB* *NM_000293.3*	Chr16-47727362C>T c.2839C > T p.(Gln947*)	rs763501714	0.000008815	Nonsense	P	Hom	GSDIXb	AR	P17
12	*PHKG2* *NM_000294.3*	Chr16-30768034C>T c.925C > T p.(Arg309Trp)	rs760257918	0.0000544	Missense	LP	Hom	GSDIXc	AR	P18, P19
13	*SLC2A2* *NM_000340.2*	Chr3-170727749CAAT>C c.491_493del p.(Tyr164del)	NA	NA	In-frame deletion	VUS	Hom	GSDXI	AR	P20

^a^ Chromosomal position according to GRCh37/hg19.

Abbreviations: AR, autosomal recessive; GSD, glycogen storage disease; Hom, homozygous; Het, heterozygous; NA, not available; P, pathogenic; LP, likely pathogenic; VUS, variant of uncertain significance.

**Fig. 1:**
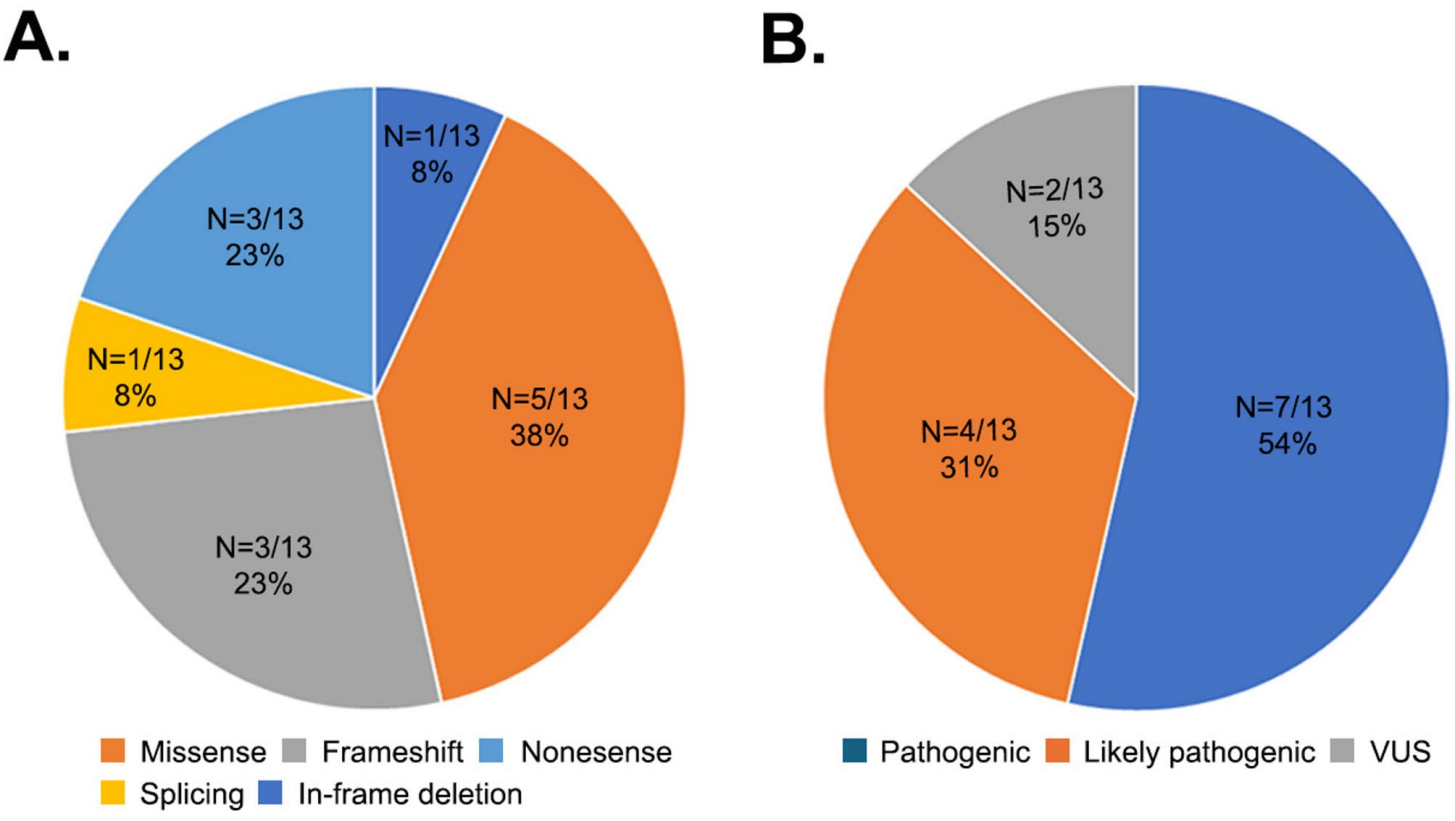
Categories and proportions of variants identified in the GSD cohort. (**A**) Type, number and proportion of identified variants. (**B**) The classification and proportion of detected variants. VUS: variants of uncertain significance

The 13 identified variants included missense (5 variants), frameshift (3 variants), nonsense (3 variants), in-frame deletion (1 variant), and a splice site (1 variant). Of these, 12 were homozygous, and 1 was hemizygous (Table 2; Fig. 1A). Four variants were identified in more than one patient. According to ACMG guidelines, 7 variants were pathogenic, 4 as likely pathogenic and 2 as variants of uncertain significance (VUS) (Fig. 1B).

In this study, we identified two variants in the *AGL* gene in two patients (P13 and P14), 3 in the *SLC37A4* gene in patients (P5-P7), 4 in the *GAA* gene in patients (P8-P12), and one in *G6PC* gene in patients (P1-P4), *GBE1* in patient (P15), *PHKA2* in patient (P16), *PHKB* in patient (P17), *PHKG2* in patients (P18 and P19), and *SLC2A2* in patient (P20) (Table 2). Among the identified variants, three detected in patients (P13, P16, and P20) have never been reported in the literature; however, they were recorded in the ClinVar database (Supplementary Table 1). These variants included *AGL* (c.1186–3C>G), *PHKA2* (c.1661G>A, and *SLC2A2* (c.491_493del). In patient (P16), we also identified a homozygous 2-base pair deletion variant (c.1173_1174del) in exon 4 of the *SLC46A1* gene, which likely explained most of his clinical symptoms.

The carrier status was confirmed by Sanger sequencing for all the probands’ parents. Genetic counselling was also offered to families seeking prenatal or preimplantation genetic diagnosis (PGD). Seven families underwent prenatal diagnosis via chorionic villus samples (CVS). Six of the families (F6, F9-F11, F15, and F20) underwent prenatal diagnosis once and one family (F12) underwent testing twice. The results revealed that two of the fetuses from families F19, and F10 were affected, four from families (F11, F12, F15, and F20) were heterozygous, and the variant was not detected in two fetuses from families F6, and F12. The family (F10) subsequently opted for PGD, which identified 2 out of 13 embryos as unaffected.

## Discussion

In this study, for the first time, we report the genetic diagnosis of GSDs within a cohort of 20 patients from Jordan. The initial diagnosis of GSD in these patients was based on clinical presentations. The metabolic screening was not performed; therefore, the definitive diagnosis of these patients was unknown. Some families had only one affected child, whereas others had multiple affected children or relatives.

We identified 13 variants in genes associated with GSDs. Importantly, we successfully identified the disease-causing variants in all patients, underscoring the diagnostic value of WES in metabolic disorders.

We identified nine different GSD types in our cohort. Pompe disease was the most common disease, accounting for 25% of the cases, followed by GSDIa (20%), GSDIb (15%), GSDIII and GSDIXC (10% each), whereas GSDIV, GSDIXa, GSDIXb, and GSDXI were each found in 5% of the patients. This distribution highlights the variability in the prevalence of different GSDs within the studied cohort.

In four patients (P1-P4), a homozygous missense variant (c.247C>T) was identified in the *G6PC* gene. This variant has been previously reported in patients with GSDIa either in homozygous or compound heterozygous states [16, 17]. Functional studies have shown that this variant results in a complete absence of glucose 6-phosphatase (G6PC) enzymatic activity, which explains the clinical manifestations of the disease [18].

The genetic diagnosis of GSDIb was confirmed in three patients (P5-P7), all harboring the same frameshift variant (c.1042_1043del) in the *SLC37A4* gene. The variant is also referred to as c.1211del in the literature and has been previously reported in both homozygous and compound heterozygous states in patients with GSDs [5, 19–21].

Pompe disease was identified in five patients (P8-P11), with four distinct variants detected in the *GAA* gene including c.716del, c.1805C>T, c.2015G>A and c.2011_2012del. The c.716del variant has been previously reported in individuals with Pompe disease in a compound heterogeneous state [22, 23]. To our knowledge, this is the first report of the homozygous state of this variant in our patient (P9).

The c.1805 C > T variant was reported in one patient (P9). This variant lies in exon 13 of the *GAA* gene and alters a conserved amino acid in the protein. In-silico tools confirmed the damaging effect of this variant. This variant has only been previously reported in compound heterozygosity with c.1726G>A, p.(Gly576Ser) in an affected individual who had slightly decreased enzymatic activity of the acid α-glucosidase (GAA) [24]. However, the c.1726G>A variant has been reported as a benign pseudodeficiency allele commonly found in healthy individuals [25–27]. Furthermore, this variant was found in a homozygous state in healthy individuals in our *in-house* exome database. The c.1805C>T variant is reported in this study, for the first time, as homozygous, emphasizing its potential pathogenicity in Pompe disease.

The frameshift (c.2011_2012del) variant was detected in patient (P10), which is predicted to trigger nonsense-mediated mRNA decay (NMD), a cellular mechanism that identifies and degrades mRNA transcripts with premature termination codons to avoid the production of nonfunctional or potentially harmful truncated proteins [28]. The identified variant has been previously reported in a Turkish child with Pompe disease [29]. Compared to our patient, who was not subjected to treatment and died at the age of 8 months, the Turkish child was under treatment and was alive at the date of genetic testing. These findings highlight the importance of early initiation of ERT in improving survival rates for affected children.

The c.2015G>A variant was found in patients (P11 and P12) from two unrelated families, both of whom displayed muscle weakness, hypotonia, and hypertrophic cardiomyopathy. This variant has been reported in multiple Pompe disease patients [30]. In this study, one family had one proband who passed away at the age of 6 months, whereas in the other family, two affected daughters who are still alive, had different responses to ERT. The younger daughter, who was treated at 3 months of age, had shown significant clinical improvement, whereas the older daughter, who began ERT at the age of 2 years, presented with muscle weakness in the lower limbs and cardiac involvement, symptoms not observed in her sister. These findings highlight the importance of early ERT in improving clinical outcomes and lifesaving [31].

GSDIII was confirmed in two patients (P13 and P14) with hepatomegaly. In patient (P13), a previously unpublished homozygous acceptor splice site variant (c.1186- C>G) in intron 9 of the *AGL* gene was identified. This patient has affected relatives with similar symptoms. This variant was confirmed by Sanger sequencing and segregated with the disease in this family. In-silico tools (SpliceAI and dbscSNV Ada) predicted altered splicing that could result in a loss-of-function (Supplemental Table 1). This variant was reported in the ClinVar database (Variant ID: 936928) as VUS. Genetic testing of the affected relatives and future functional analysis at the RNA level will be helpful in confirming the pathogenicity of this variant. On the other hand, the outcome of the two prenatal tests for this family revealed that one fetus was a carrier whereas in the second pregnancy, the variant was not detected, which further supports the disease-causing effect of this variant.

In one patient (P14), a homozygous nonsense variant (c.2929C>T) was identified in the *AGL* gene. This variant is predicted to cause a premature stop codon (p.(Arg977*)) likely resulting in loss-of-function. This variant has been reported six times in gnomAD (allele frequency 0.00000411) and is classified as likely pathogenic/pathogenic in the ClinVar database (variant ID 370992). Previous studies have reported this variant only in a heterozygous state with other *AGL* variants in GSD III patients [32–34]. For the first time, we report, the homozygous state of this variant.

GSDIV was confirmed in one patient (P15) who presented with hepatosplenomegaly. A biallelic missense variant (c.986A>G) was identified in the *GBE1* gene. This variant has been previously reported in a compound heterozygous state in individuals with adult polyglucosan body disease [35], and as homozygous or heterozygous (without a reported second allele) in GSDIV patients [36, 37]. Furthermore, the variant has been reported as likely pathogenic for GSDIV but as a VUS for adult polyglucosan body disease (ClinVar: Variation ID: 371439). On the other hand, other missense variants at the same codon (Y329S and Y329F) have been identified in patients with either GSDIV or adult polyglucosan body disease [35, 38], highlighting the functional importance of the Y329 residue in the GBE1 protein and supporting the association of this variant with the disease.

The GSDIXa was found in one patient (P16) with a hemizygous nonsense variant (c.1661G>A) in the *PHKA2* gene. This variant is predicted to cause premature protein termination (p.(Trp554*)), resulting in the loss of calmodulin-binding regions, which likely leads to loss of function. This variant has not been reported in population databases (gnomAD, 100 genomes and ExAC) or the literature; however, it has been documented in ClinVar as pathogenic for GSDIXa1 (Variants ID: 3255575). Given that loss-of-function variants in the *PHKA2* gene are known to cause the disease [39, 40], this variant can be classified as pathogenic according to ACMG guidelines (Supplementary Table 1).

Our patient had severe diarrhea, and severe abdominal distention, which was consistent with GSDIXa. However, additional symptoms unrelated to GSDs including fever, neutropenia, thrombocytopenia, dysplastic bone marrow, and low serum folic acid as well as a history of blood transfusion, were also observed, suggesting the presence of another condition. The reanalysis of the patient’s WES revealed another homozygous 2-base pair deletion (c.1173_1174del) in the *SLC46A1* gene (ClinVar: Variants ID: 3255576), which likely explains the observed symptoms. This likely pathogenic variant is predicted to cause a frameshift and premature protein termination (p.(Ser393CysfsTer7)).

Notably, common features of GSDIXa such as hypoglycemia and hepatomegaly were not observed in this patient at the time of diagnosis. Unfortunately, we were unable to communicate with the patient’s family for further follow-up. Based on these genetic results and clinical presentations, this patient is most likely to have two diseases and GSDIXa may develop later in life [41].

In this study, we identified a previously reported homozygous pathogenic nonsense variant (c.2839 C > T) in the *PHKB* gene in one patient (P17) [42] who had elevated liver enzymes, similar to our patient in addition to a marked reduction in phosphorylase kinase (PhK) enzyme activity, which confirms the diagnosis of GSDIXb.

GSDIXc was confirmed in two patients (P18 and P19) who manifested similar symptoms. A known homozygous likely pathogenic missense variant (c.925C>T) was detected in the *PHKG2* gene [43, 44].

In the last patient (P20), a homozygous in-frame deletion (c.491_493del) in the *SLC2A2* gene was identified. This variant results in the deletion of a single codon leading to the loss of a tyrosine at position 164 (p.(Tyr164del)). However, this variant has not been previously reported in literature or in gnomAD, 100 Genomes and ExAC, it is listed as a VUS in the ClinVar database (Variant ID: 2436012) in the context of Fanconi-Bickel syndrome (GSDXI). A prenatal diagnosis was later performed for this family, which revealed an unaffected fetus (heterozygous). The child is now 2 years old and healthy. Sanger sequencing confirmed the carrier status of both parents. Based on these findings, the classification of this variant can be upgraded to likely pathogenic.

As a part of disease prevention, we performed 8 prenatal diagnoses for 7 families. Genetic testing revealed that two of the fetuses were affected, and following genetic counseling, these pregnancies were terminated. Moreover, one of these families later opted for PGD where 2 out of 13 embryos were found to be normal.

In this study, we were unable to communicate with some families, which prevented patients’ follow-up. While genetic testing is highly effective in providing rapid and accurate diagnoses for genetic diseases including GSDs, additional biochemical testing is crucial to confirm the pathogenicity of the identified variants, particularly when dealing with novel or VUS variants.

## Conclusions

We identified disease-causing variants in 20 GSD patients, which further expands the spectrum of genetic variations associated with GSDs. The high consanguinity rate within families contributes to the occurrence of these disorders. Our findings will, therefore, be valuable for genetic counseling, prenatal diagnosis and PGD. This study demonstrated that WES is an efficient and cost-effective diagnostic tool for GSDs. Accurate genetic diagnosis not only facilitates the identification of GSD subtypes but also enables early intervention and more effective disease management.

## Supplementary Information

Below is the link to the electronic supplementary material.

Supplementary Material 1

## Data Availability

The datasets used and/or analyzed during the current study are available from the corresponding author on reasonable request.

## Abbreviations

WES: Whole exome sequencing
VUS: Variant of uncertain significance
GSD: Glycogen storage diseases
PND: Prenatal diagnosis
PGD: Preimplantation genetic diagnosis
ERT: Enzyme replacement therapy
