# Supplementary material for: Genetic diagnosis of Jordanian patients with glycogen storage diseases
Source: Orphanet J Rare Dis. 2025 Oct 3;20:501. doi: 10.1186/s13023-025-03982-z (PMC12495632; doi:10.1186/s13023-025-03982-z)
Supplement: Supplementary file 1 — Supplementary Material 1 [file 13023_2025_3982_MOESM1_ESM.docx]

| Supplementary Table 1: Pathogenicity assessment of the three unpublished variants. | | | | | | | | | | | | | | |
| --- | --- | --- | --- | --- | --- | --- | --- | --- | --- | --- | --- | --- | --- | --- |
| # | **Patient** | **Gene RefSeq** | **Variant** | **gnomAD AF** | **Mutation Taster** | **Mutation Assessor** | **Revel** | **CADD** | **SiPhy** | **dbscSNV Ada** | **Splice AI** | **ClinVar (Accession)** | **ACMG criteria** | **ACMG** |
| 1 | P13 | *AGL* NM_000152.5 | c.1186-3C>G | NA | NA | NA | NA | 16.99 | NA | 1.0 (D) | 0.59 (DS) | VCV000936928.9 | PM2_Supporting PP3_Strong | VUS |
| 2 | P16 | *PHKA2* NM_000292.3 | c.1661G>A (p.Trp554*) | NA | 1.0 (DC) | NA | NA | 27.5 | NA | NA | NA | VCV003255575.2 | PVS1_Strong PM2_Supporting PP5_Supporting | P |
| 3 | P26 | *SLC2A2* NM_000340.2 | c.491_493del (p.Tyr164del) | NA | NA | NA | NA | NA | NA | NA | NA | VCV002436012.3 | PM2_Supporting PM4_Moderate | VUS |
| Abbreviations: AF, allele frequency; D, deleterious; Dam, damaging; DC, disease-causing; DS, Disruption of a splice site; H, high; P, pathogenic; LP, likely pathogenic; NA, not available; VUS, variant of uncertain significance. | | | | | | | | | | | | | | |
